# Raw milk cheeses from Beira Baixa, Portugal—A contributive study for the microbiological hygiene and safety assessment

**DOI:** 10.1007/s42770-024-01332-y

**Published:** 2024-04-15

**Authors:** Rita Mendonça, Rosália Furtado, Anabela Coelho, Cristina Belo Correia, Elena Suyarko, Vítor Borges, João Paulo Gomes, Angela Pista, Rita Batista

**Affiliations:** 1 Department of Food and Nutrition, National Institute of Health Doutor Ricardo Jorge, Lisbon, Portugal; 2 Faculty of Sciences, University of Lisbon, Lisbon, Portugal; 3 Department of Infectious Diseases, National Institute of Health Doutor Ricardo Jorge, Lisbon, Portugal; 4 NOVA School of Science and Technology, 2829-516 Caparica, Portugal; 5 Animal and Veterinary Research Center (CECAV), Faculty of Veterinary Medicine, Lusófona University-Lisbon University Centre, Lisbon, Portugal

**Keywords:** Raw milk cheese, *Listeria monocytogenes*, *Escherichia coli*, Coagulase Positive Staphylococci, *Salmonella* spp., Whole-genome sequencing (WGS), Portugal

## Abstract

Due to specific bacterial microbiota, raw milk cheeses have appreciated sensory properties. However, they may pose a threat to consumer safety due to potential pathogens presence. This study evaluated the microbiological contamination of 98 raw milk cheeses from Beira Baixa, Portugal. Presence and enumeration of Coagulase Positive Staphylococci (CPS), *Listeria monocytogenes*, *Salmonella* spp., pathogenic *Escherichia coli*, and indicator microorganisms (non-pathogenic *E. coli* and *Listeria* spp.) was attained. *E. coli* antimicrobial resistance (AMR) was also evaluated. PCR and/or Whole genome sequencing (WGS) was used to characterize *E. coli*, *Salmonella* spp. and *L. monocytogenes* isolates. Sixteen cheeses (16.3%) were classified as Satisfactory, 59 (60.2%) as Borderline and 23 (23.5%) as Unsatisfactory/Potential Injurious to Health. *L. monocytogenes*, CPS > 10^4^ cfu g^−1^, Extraintestinal pathogenic *E. coli* (ExPEC) and *Salmonella* spp. were detected in 4.1%, 6.1%, 3.1% and 1.0% of the samples, respectively. *Listeria innocua* (4.1%) and *E. coli* > 10^4^ cfu g^−1^ (16.3%) were also detected. AMR *E. coli* was detected in 23/98 (23.5%) of the cheese samples, of which two were multidrug resistant. WGS identified genotypes already associated to human disease and *Listeria* spp. cluster analysis indicated that cheese contamination might be related with noncompliance with Good Hygiene Practices during cheese production.

**Supplementary Information:**

The online version contains supplementary material available at 10.1007/s42770-024-01332-y.

## Introduction

Traditional cheeses are dairy products characteristic of a certain geographic region. These cheeses may be made from raw milk and the method of production is passed down from generation to generation. The microbiota of raw milk is complex, derived from many sources (e.g. microorganisms from teats, the farm environment, feedstuffs, as well as milking and processing equipment) and can have both positive and negative impacts on the cheese quality and shelf life and, consequently, on its economic potential. The diverse indigenous microbiota of raw milk cheeses provide fermentation, contribute to ripening and is responsible for the specific sensory properties of raw milk cheeses, namely a more intense and stronger flavor [1–3]. Also, some authors suggest a beneficial link between the consumption of raw milk microbes, and protection against the development of asthma and atopy, later in life [4, 5] and the reduction of the blood pressure in people with mild to moderate hypertension [6]. However, raw dairy products can be contaminated with pathogens and consequently may have health-related implications leading to severe illnesses [7]. In fact, estimates by the Center for Disease Control and Prevention, in the USA, based on data collected between 1993 and 2006, suggest that non pasteurized milk and milk products caused a disproportionate number of outbreaks and outbreak-associated illnesses relative to consumption of pasteurized products (≈150 × greater/unit of product consumed) [8]. Furthermore, Costard *et al.*, made a study on raw milk and cheese data from 2009 to 2014 and estimated that consumption of these raw products causes 840 times more illnesses and 45 times more hospitalizations than pasteurized products [9].

Microbial contamination during cheese-making, ripening and storage may occur directly or through cross contamination events, during processing, in retail or in domestic environments [10]. Although the microbiological quality and safety of raw milk cheeses begin with milk, which may be the primary source of contamination [11], other sources of contamination can be present. Indeed, in the production environment, in food-contact surfaces and utensils that are not properly clean and sanitized some pathogenic microorganisms are able to adhere and persist forming biofilms [12]. The hands of the workers, and the unsafe handling and inadequate storage practices in domestic environments [13] are also potential sources of cheese contamination.

According to the European Food Safety Authority (EFSA) [14], between 2018 and 2022, milk and milk products caused 149 strong evidence outbreaks, with 1754 human cases, 298 hospitalizations and 22 deaths, on Europe [14]. *Salmonella* spp. was the leading causative agent being responsible for 27.52% of the outbreaks, *Staphylococcus aureus* toxins were associated with 15.44% and Shiga-toxin producing *E. coli* (STEC) was detected in 6.04% of these 149 outbreaks. *L. monocytogenes*, although only associated with 4.03% of the outbreaks, was responsible for a high number of deaths, 14 of the 22 reported [14].

Although raw milk cheeses are niche products on the global market, they constitute an important fraction of the cheese production in Portugal and are part of Portuguese cultural and gastronomic identity [15]. The protection and promotion of these products is essential as they help to reduce rural depopulation, develop existing resources and generate employment opportunities, among other benefits. Some of the Portuguese raw milk cheese brands (n = 11) have a registration of Protected Designation of Origin (PDO), which means that they have special characteristics related to the geographical place in which they were produced/ processed [16]. Beira Baixa is a historically important geographical area in terms of cheese production, and is one of the Portuguese demarcated regions for this artisanal activity. The geographical area of Beira Baixa cheeses production covers the municipalities of Castelo Branco district and part of the Santarém district, in the central region of Portugal. The type of rennet (of animal or of vegetal origin- *Cynara Cardunculus*) and the temperature, relative humidity and duration of the maturation period vary between brands. Beira Baixa cheeses with PDO label are “Castelo Branco”, “Amarelo da Beira Baixa” and “Picante da Beira Baixa”. “Castelo Branco” PDO labeled cheese is a semi-hard or semi-soft paste cured cheese with a yellowish color produced with ewe’s raw milk; “Amarelo da Beira Baixa” is a semi-hard or semi-soft paste cured cheese with a yellowish color produced with ewe’s or ewe’s and goat’s raw milk and “Picante da Beira Baixa” is a semi-hard to hard paste cured cheese with a greyish white color, produced with ewe’s and goat’s raw milk. Beira Baixa PDO labeled cheeses have a ripening period of at least 45 days.

The goal of this study was to contribute to the microbiological contamination assessment of cured raw milk cheeses produced in Beira Baixa region, Portugal. This assessment could potentially contribute to improve the implemented safety systems and consequently the quality of the cheeses in the studied region.

## Materials and methods

### Sampling

In this study, a total of 98 cured raw milk cheeses from Beira Baixa region, Portugal, were analyzed, corresponding to 32 different brands produced by 9 producers (Supplementary Table 1). All samples came from different batches, and were purchased in several retail establishments, nearby Lisbon area, from October 2022 to June 2023. Samples were kept under refrigeration conditions (2 °C to 4 °C) from purchase until analysis, which occurred within a maximum of 24 h after purchase and within their assigned shelf-life period.

### Microbiological analysis

ISO 7218: 2007 general requirements and guidance for microbiological examinations [17] were followed for all microbiological analysis.

The 98 cheese samples were analyzed for the presence and enumeration of some common foodborne pathogens such as Coagulase Positive Staphylococci (CPS*), Listeria monocytogenes*, *Salmonella* spp. and pathogenic *Escherichia coli*, as well as of indicator microorganisms (non-pathogenic *E. coli* and *Listeria* spp. other than *L. monocytogenes*). Staphylococcal enterotoxins (SE) detection was also assessed for those samples with a CPS concentration ≥ 4.9 × 10^4^ cfu/g.

#### *Salmonella* spp. detection and *E. coli* and Coagulase Positive Staphylococci (CPS) detection and enumeration

Sample preparation was performed as followed: 25 g of each cheese sample was homogenized, at 230 rpm for 1 min using a stomacher (Stomacher, 400 Circulator, London, UK), in a sterile bag with 225 mL of Buffered peptone water (BPW-Oxoid, Basingstoke, Hampshire, UK), as described on ISO 6579–1: 2017 protocol [18].

For detection and enumeration of *E. coli* and CPS, the AFNOR validated TEMPO® EC (BIO12/13–02/05) and TEMPO® STA (BIO12/28–04/10) automated most probable number (MPN) system (bioMérieux, Marcyl l’Etoile, France) were used, respectively, according to the manufacturer’s instructions. For these analyses, 1 mL of decimal dilutions 10^–1^ and 10^–3^ of the primary mixture, in tryptone salt diluent (Biokar Diagnostics, Pantin, France), were used. The remaining mixture was incubated at 37 °C ± 1 °C for 18 h ± 2 h (non-selective pre-enrichment) and used for the detection of *Salmonella* spp. and for plating out of *E. coli*. For the detection of *Salmonella* spp., 0.1 mL of the incubated non-selective pre-enrichment was transferred into 10 mL of Salmonella Xpress 2 (SX2) broth (bioMérieux), incubated at 41.5 °C ± 1 ºC, for 24 h ± 2 h and VIDAS® Easy SLM (bioMérieux) AFNOR validated method (BIO-12/16–09/05) was used, according to the manufacturer’s instructions. For those samples positive for *Salmonella* spp., in VIDAS, a drop of the SX2 enrichment, was streaked on IRIS Salmonella agar (BIOKAR Diagnostics) and another one on xylose lysine deoxycholate (XLD, bioMérieux) agar and incubated at 37 °C ± 1 ºC, for 24 h ± 3 h.

For *E. coli* plating out, a loopful of the incubated non-selective pre-enrichment was streaked on the surface of Chromogenic Coliform Agar (CCA, Biokar Diagnostics) plates, and incubated at 37 °C during 24 h ± 2 h. Hemolytic activity of presumptive *E. coli* colonies was tested by sub-culture on Columbia Agar + 5% Sheep Blood (COS; bioMérieux) and incubation at 37 °C during 24 h ± 2 h. The presumptive *E. coli* and *Salmonella* spp. isolates were confirmed on VITEK®2 compact system (bioMérieux) and all positive isolates stored at -80 °C in broth with 20% glycerol.

#### Detection and enumeration of *Listeria* spp.

For *L. monocytogenes* detection, 25 g of each cheese sample was added to 225 ml of half Fraser broth (bioMérieux), as the primary enrichment culture, in a stomacher bag and were homogenized in a stomacher for 1 min and incubated for 24 ± 1 h at 30 °C. One hundred microliters of the primary enrichment culture, was then added to 10 mL of Fraser broth (bioMérieux), as a second enrichment culture, and incubated at 37 °C for 24 h ± 2 h [19]. After incubation, 0.5 mL of the second enrichment culture was tested using the AFNOR validated VIDAS® LMO2 automated method (BIO12/11–03/04), according to the manufacturer’s instructions.

*L. monocytogenes* enumeration was performed according to ISO/11290–2 horizontal method [20]. Briefly, 10 g of each VIDAS® LMO2 positive sample was added to 90 mL of BPW and homogenised (Initial Suspension). *L. monocytogenes* presumptive colonies were counted after the spread of 1 mL of the Initial Suspension on the surface of Microinstant® Listeria Agar (Ottaviani e Agosti) (Biokar Diagnostics) plates and incubation at 37 °C for 48 h ± 2 h. *L. monocytogenes* presumptive colonies (blue colored surrounded by an opaque halo) as well as *Listeria* spp., other than *L. monocytogenes* (blue colonies without an opaque halo) were isolated on Columbia Agar + 5% Sheep Blood (COS; bioMérieux) at 37 °C for 24 h ± 2 h, where hemolytic activity was assessed. Confirmation of the identification of the isolates was attained on VITEK®2 compact system (bioMérieux), following the manufacturer’s instructions. All positive isolates were stored in Tryptone Soy Broth (TSB; Biokar Diagnostics) with 20% glycerol, at -80 °C.

#### Detection of Staphylococcal Enterotoxins (SE)

All cheese samples presenting Coagulase Positive Staphylococci levels ≥ 4.9 × 10^4^ cfu/g were tested for the presence of staphylococcal enterotoxins (SE), as described on ISO 19020:2017 [21]. In summary, the first step consisted in an extraction where toxin diffusion was attained by adding 25 g of each tested cheese sample to 40 mL of distilled water at 37 °C ± 1 °C and, after homogenization, the mixture was shaken for 30–60 min at room temperature in an VXR basic Vibrax orbital shaker (Ika®,Staufen, Germany). The pH was adjusted, the mixture centrifuged and then the resulting supernatant was concentrated by dialysis with a 6000–8000 Da molecular cut-off membrane (Spectrum Laboratories,Rancho Dominguez, CA, USA) against 30% (w/v) of polyethylene glycol 20,000 (Merck,Darmstadt, Germany), overnight, at 4 °C. Finally, immunoenzimatic detection was performed using the automated method VIDAS® Staph enterotoxin II (SET 2) (bioMérieux).

### Microbiological results interpretation and statistical analysis

Microbiological results were interpreted according to the criteria showed on Table 1. The used criteria was based on the National Institute of Health Doutor Ricardo Jorge (INSA) guidelines for the interpretation of microbiological assays [22], the Commission Regulation (EC) Nº 2073/2005 [23], the Health Protection Agency- Guidelines for assessing the Microbiological Safety of Ready-to-eat Foods Placed on the market [24] and on the Luxembourg Microbiological criteria applicable to foodstuffs [25].

**Table 1:** Criteria used for microbiological safety classification of the samples

Microbiological Safety Interpretation	Parameters					
	Pathogens				Indicator microorganisms	
	*L. monocytogenes*^a^ (cfu g^−1^)	*Salmonella* spp.^b^	CPS^c^ (cfu g^−1^)	STEC and non-STEC^d^	*E. coli*^e^ (cfu g^−1^)	*Listeria* spp*. (*not *L. monocytogenes)*^f^ (cfu g^−1^)
Satisfactory	ND	ND	< 10	ND	< 10	< 10
Borderline	≤ 10^2^	N/A	10—≤ 10^4^	N/A	10- ≤ 10^4^	10- ≤ 10^2^
Unsatisfactory/ Potential Injurious to Health	> 10^2^	D	> 10^4^	D	> 10^4^	> 10^2^

**cfu/g**- colony forming units *per* gram; **STEC**- Shiga Toxin-producing *Escherichia coli;* **CPS**- Coagulase Positive Staphylococci; **ND** – Not detected in 25 g; **D** – Detected in 25 g; **N/A**- Not applicable; ^a,b,c^- in accordance with [22–24, 25]; ^d,f^- in accordance with [22, 24]; ^e^- in accordance with [25]

A cheese brand obtained a Satisfactory microbiological quality classification when all the samples of that brand were classified as Satisfactory for all the tested parameters. Borderline classification was attained when at least one of the tested samples was classified as Borderline, in at least one of the tested parameters, and none of them was classified as Unsatisfactory, in any of the tested parameters. Unsatisfactory/Potential Injurious to Health (U/PIH) classification was attributed when at least one sample was classified as Unsatisfactory/Potentially Injurious to Health, in at least one of the tested parameters.

Fisher- Freeman- Halton’s exact test of independence, with a 99.0% degree of confidence, was performed, using IBM SPSS Statistics 27.0.1 software, to determine if there was a significant relationship between the independent variables “microbiological safety classification” and the type of milk used in cheese production (“presence of cow’s milk”, “presence of ewe’s milk”, “presence of goat’s milk”) and between “microbiological safety classification” and registration of “Protected Designation of Origin (PDO)”.

### Antimicrobial susceptibility testing of *E. coli* and *Salmonella* spp. isolates, *Salmonella* spp. serotyping and pathogenic *E. coli* identification

Since the use of antimicrobial agents in animal farming is considered as one of the most critical factors that contribute to the emergence and dissemination of antibiotic resistant bacteria, and because *E. coli* is considered a potential indicator of antimicrobial resistance (AMR), *E. coli* isolates (pathogenic and non-pathogenic) AMR was evaluated.

*E. coli* antimicrobial susceptibility testing (AST) was performed on 91 strains, isolated from 91 cheese samples, using a panel of 17 antimicrobials (Amoxicillin-Clavulanic Acid, Ampicillin, Azithromycin, Cefepime, Cefotaxime, Cefoxitin, Ceftazidime, Ceftriaxone, Chloramphenicol, Ciprofloxacin, Gentamicin, Meropenem, Nalidixic Acid, Sulfamethoxazole, Tetracycline, Tigecycline, Trimethoprim), following the Kirby-Bauer method and the European Committee on Antimicrobial Susceptibility Testing recommendations (EUCAST) [26].

For *Salmonella* spp. isolate, 19 antimicrobials were tested (Amikacin, Amoxicillin-Clavulanic Acid, Ampicillin, Azithromycin, Cefepime, Cefotaxime, Cefoxitin, Ceftazidime, Ceftriaxone, Chloramphenicol, Gentamicin, Kanamycin, Meropenem, Nalidixic Acid, Pefloxacin, Sulfamethoxazole, Tetracycline, Tigecycline, Trimethoprim), following the same method and recommendations [26].

An isolate was considered as multidrug resistant (MDR) when presenting resistance to three or more antimicrobial classes [27].

*Salmonella* spp. was serotyped by the slide agglutination method for O and H antigens (SSI, Copenhagen, Denmark), according to the Kauffmann-White-Le Minor scheme [28].

The pathogenicity of the *E. coli* isolates was assessed by the detection of intestinal pathotype- specific virulence genes (*eae*, *aggR*, *aaiC*, *aatA*, *elt*, *esth*, *estp*, *ipaH*, *stx1* and *stx2*) as previously described [29].

The detection of at least one of the pathotype-specific genes in each isolate allowed the classification of potentially pathogenic (STEC, Shiga Toxin-producing *E. coli*; EAEC, Enteroaggregative *E. coli*; EPEC, Enteropathogenic *E. coli*; ETEC, Enterotoxigenic *E. coli*; EIEC, Enteroinvasive *E. coli*). Furthermore, *E. coli* pathogenicity was also inferred by Whole Genome Sequencing (WGS), since some of the likely non-pathogenic *E. coli* isolates were MDR and/or hemolytic, and were classified as Extraintestinal pathogenic *E. coli* (ExPEC). In these cases, the presence of two or more typical virulence genes allowed the ExPEC classification [30].

### *Listeria* spp., *Salmonella* spp. and *E. coli* whole-genome sequencing, in Silico typing and screening of *E. coli* virulence/AMR genes

Extraction of genomic DNA from all MDR and hemolytic *E. coli* as well as from all *Listeria innocua*, *L. monocytogenes* and *Salmonella* spp. isolates was achieved using the ISOLATE II Genomic DNA Kit (Bioline, London, England, UK). Extracted DNA was quantified, with the dsDNA HS Assay Kit (Thermo Fisher Scientific, Waltham, MA, USA), in the Qubit fluorometer (Invitrogen, Waltham, MA, USA), according to the manufacturer’s instructions. NexteraXT library preparation protocol (Illumina, San Diego, CA, USA) was used for DNA preparation for sequencing. Cluster generation and sequencing (2 × 150 bp) were performed on either a MiSeq, a NextSeq 550 or NextSeq 2000 instrument (Illumina).

Regarding *Listeria* spp., we performed read quality control, trimming and de novo genome assembly with the INNUca pipeline v4.2.2 (https://github.com/B-UMMI/INNUca) [31], using default parameters. In brief, FastQC v0.11.5 (http://www.bioinformatics.babraham.ac.uk/projects/fastqc/) and Trimmomatic v0.38 [32] were used for reads quality control and improvement, and de novo assembly was perfomed with SPAdes v3.14 [33]. Bowtie2 v2.2.9 [34] and Pilon v1.23 [35] were applied for final assembly curation. Kraken2 v2.0.7 [36] was used for the screening of species confirmation/contamination and mlst v2.18.1 (https://github.com/tseemann/mlst) for Sequence Type (ST) determination.

FastQ files of each *E. coli* and *Salmonella* spp. isolates were uploaded on Enterobase QAssembly pipeline, v3.61 (https://enterobase.warwick.ac.uk/species/ecoli/upload_reads and https://enterobase.warwick.ac.uk/species/senterica/upload_reads, respectively) where trimming was achieved with Sickle v1.33, de novo genome assembly with SPADES v3.9.0, assembly polish with BWA 0.7.12-r1039 and species confirmation with Kraken.

*E. coli* and *Salmonella* spp. assemblies were uploaded on the Center for Genomic Epidemiology web services (http://www.genomicepidemiology.org/services/) to determine the presence of *E. coli* virulence genes (VirulenceFinder 2.0), *Salmonella* spp. in silico serotyping (SeqSero 1.2), *E. coli *in silico serotyping (SerotypeFinder 2.0), antimicrobial resistance genes (ResFinder 4.1), and in silico Multilocus Sequence Typing (MLST) (MLST 2.0).

Sequencing reads were deposited on the European Nucleotide Archive (ENA) under the bioprojects PRJEB31216 (*Listeria* spp.), PRJEB54735 (*E. coli*) and PRJEB32515 (*Salmonella* spp.), as well as on EFSA WGS portal. Supplementary Table 1 presents the accession numbers for each isolate.

### Core-Genome clustering analysis of *Listeria* spp.

For *L. monocytogenes*, allele-calling was performed over the INNUca polished genome assemblies with chewBBACA v2.8.5 [37] using the core-genome Multi Locus Sequence Typing (cgMLST) 1,748-loci Pasteur schema [38] available at Chewie-NS website (https://chewbbaca.online/, downloaded on June 23rd, 2022) [39]. The cgMLST clustering analysis was performed with ReporTree v.2.0.3 (https://github.com/insapathogenomics/ReporTree) [40] using GrapeTree (MSTreeV2 method) [41], with clusters of closely related isolates being determined and characterized at a distance thresholds of 1, 4, 7 and 15 allelic differences (ADs). A threshold of seven ADs can provide a proxy to the identification of genetic clusters with potential epidemiological concordance (i.e., “outbreaks”) [42].

For *L. innocua*, in the absence of a cgMLST schema, a core-genome alignment of the INNUca polished assemblies was constructed with Parsnp v.1.7.4 implemented on Harvest suite [43], using the default parameters, with exception of parameter –C, which was adjusted to 2000 in order to maximize the resolution. The core-genome SNP-based clustering analysis was performed with ReporTree v.2.0.3 (https://github.com/insapathogenomics/ReporTree) [40] using GrapeTree (MSTreeV2 method) [41], with clusters of closely related isolates being determined and characterized at a SNP thresholds of 1, 4, 7 and 15 SNPs. This core-genome SNP-based clustering analysis relied on a core-genome alignment (comprising 95% of the *L. innocua* genome size) involving a total 33,081 variant sites. Interactive phylogenetic tree visualization was conducted with GrapeTree [41].

## Results

### Microbiological safety of the samples

**Fig. 1:**
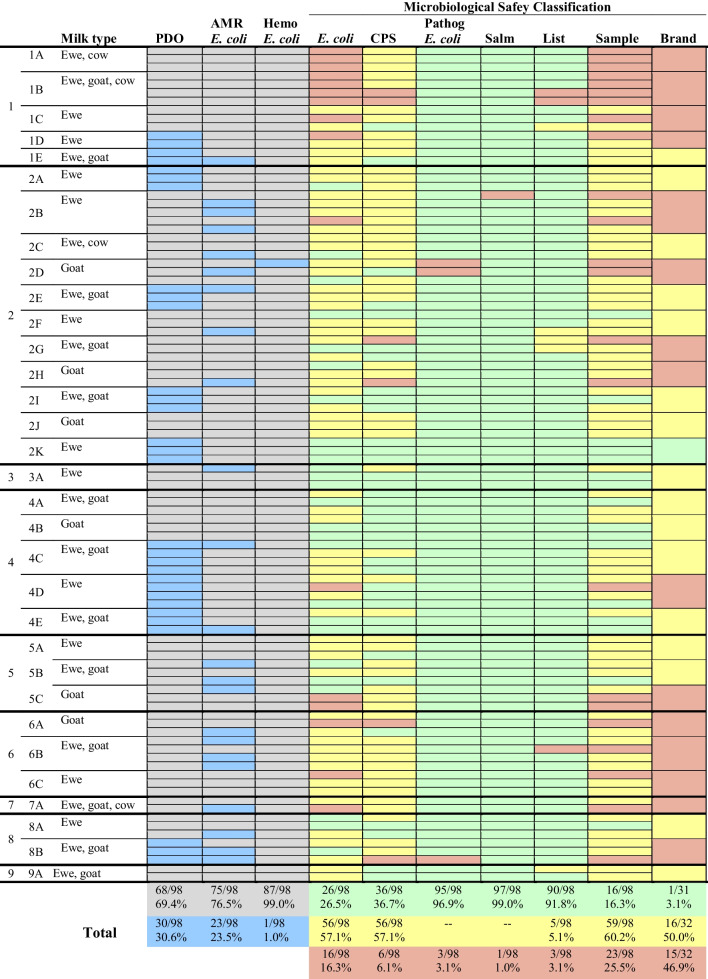
Microbiological results of the 98 tested cheeses, by producer (*N* = 9; 1–9) and brand (*N* = 32; 1A-9A). In the three first columns of the heatmap- like figure, the code Blue for Yes and Grey for No was used to inform about the existence of a Protected Designation of Origin (PDO) for each sample, the isolation of an *E. coli* with antimicrobial resistance (AMR) to at least one of the tested antimicrobials, and the detection of an hemolytic (Hemo) *E. coli*. The Green for Satisfactory; Yellow for Borderline and Red for Unsatisfactory/ Potential Injurious to Health code was used to characterize the Microbiological Safety classification of each of the 98 samples regarding the tested parameters (*E. coli*, Coagulase Positive Staphylococci (CPS), pathogenic (pathog) *E. coli*; *Salmonella* spp. (Salm) and *Listeria* spp. (List). In the last two columns of the heatmap, the global Microbiological Safety classification of each sample and brand are presented

From a total of 98 tested cheeses, 16 (16.3%) were classified as Satisfactory, 59 (60.2%) as Borderline and 23 (25.5%) as Unsatisfactory/ Potential Injurious to Health (Fig. 1, Supplementary Table 1). *L. monocytogenes* was detected in 4/98 (4.1%) of the samples, three of which in a level > 100 cfu/g. *L. innocua* was also detected in 4/98 (4.1%) of the samples. In 16 samples, *E. coli* was present in levels > 10^4^ cfu/g (16.3%) and in 3/98 (3.1%) Extraintestinal pathogenic *E. coli* (ExPEC) was identified. *Salmonella* spp. was detected in one sample (1.0%) and CPS > 10^4^ cfu/g was detected in 6/98 (6.1%) of the samples (Fig. 1, Supplementary Table 1). Staphylococcal enterotoxins (SE) were not detected in any of the analyzed samples.

The classification Unsatisfactory/Potential Injurious to Health (U/PIH) on 13 samples, was exclusively due to the enumeration of *E. coli* > 10^4^ cfu/g (13/23; 56.5%). The other 10 samples were classified as U/PIH due to the presence of CPS > 10^4^ cfu/g (2/23; 8.7%); *L. monocytogenes* > 10^2^ cfu/g (1/23; 4.4%); ExPEC (2/23; 8.7%); *E. coli* and CPS > 10^4^ cfu/g and *L. monocytogenes* > 10^2^ cfu/g (2/23; 8.7%); CPS > 10^4^ cfu/g and ExPEC (1/23; 4.4%); *E. coli* and CPS > 10^4^ cfu/g (1/23; 4.4%); and finally, one cheese (1/23; 4.4%) was classified as U/PIH because *Salmonella* spp. was detected in 25 g (Fig. 1, Supplementary Table 1).

When focusing on brands’ microbiological safety, from the 32 analyzed brands, 15 (15/32; 46.9%) were categorized as U/PIH, 16 (16/32; 50.0%) as Borderline and only one (1/32; 3.1%) as Satisfactory (Fig. 1). With the exception of producers 3 and 9, for which only one brand was analyzed (classified as Borderline), all the other producers presented always some brands classified as U/PIH (Fig. 1).

When looking at the obtained results considering the type of milk used in cheese production, it was possible to find a statistical significant association between microbiological safety classification and the presence of cow’s milk (Fisher-Freeman-Halton = 11.785, p = 0.001) (Table 2). There was a clear decrease in the number of Satisfactory samples and an increase of Unsatisfactory/Potentially Injurious to Health samples, when cheese samples contained cow’s milk (Table 2). This association occurred also for microbiological safety classification regarding Coagulase Positive Staphylococci and *E. coli* enumeration and the presence of cow’s milk in the cheese samples (Table 2). There was no statistical significant association between microbiological safety classification and the presence of ewe’s or goat’s milk, nor statistical significant association between microbiological safety classification and registration of Protected Designation of Origin (Table 2). There was also no statistical significant association between microbiological safety classification of the cheese samples and the number of types of milk used (Fisher-Freeman-Halton = 10.250, p = 0.025) (data not shown).

**Table 2:** Fisher-Freeman-Halton’s exact test results- statistical association between Microbiological Safety classification and the type of milk used in cheese production and between Microbiological Safety classification and registration of Protected Designation of Origin (PDO)

		Presence of cow’s milk				Fisher-Freeman-Halton	*p*-value
		No		Yes		11.785	0.001
		Observed	Expected	Observed	Expected		
MS	Satisfactory	16	14.0	0	2.0		
	Borderline	55	51.8	4	7.2		
	U/PIH	15	20.2	8	2.8		
	Total = 98	86		12			
		Presence of cow’s milk				Fisher-Freeman-Halton	*p*-value
		No		Yes		10.624	0.004
		Observed	Expected	Observed	Expected		
MS (CPS)	Satisfactory	36	31.6	0	4.4		
	Borderline	46	49.1	10	6.9		
	U/PIH	4	5.3	2	0.7		
	Total = 98	86		12			
		Presence of cow’s milk				Fisher-Freeman-Halton	*p*-value
		No		Yes		18.075	5.9e^−5^
		Observed	Expected	Observed	Expected		
MS (EC)	Satisfactory	25	22.8	1	3.2		
	Borderline	53	49.1	3	6.9		
	U/PIH	8	14.0	8	2.0		
	Total = 98	86		12			
		Presence of ewe’s milk				Fisher-Freeman-Halton	*p*-value
		No		Yes		1.313	0.536
		Observed	Expected	Observed	Expected		
MS	Satisfactory	2	2.9	14	13.1		
	Borderline	10	10.8	49	48.2		
	U/PIH	6	4.2	17	18.8		
	Total = 98	18		80			
		Presence of goat’s milk				Fisher-Freeman-Halton	*p*-value
		No		Yes		0.590	0.808
		Observed	Expected	Observed	Expected		
MS	Satisfactory	8	6.7	8	9.3		
	Borderline	24	24.7	35	34.3		
	U/PIH	9	9.6	14	13.4		
	Total = 98	41		57			
		Protected Designation of Origin				Fisher-Freeman-Halton	*p*-value
		No		Yes		6.240	0.041
		Observed	Expected	Observed	Expected		
MS	Satisfactory	8	11.1	8	4.9		
	Borderline	40	40.9	19	18.1		
	U/PIH	20	16.0	3	7.0		
	Total = 98	68		30			

**MS-** Microbiological Safety Classification*;* **CPS**- Coagulase Positive Staphylococci; **EC**- *E. coli;* **U/PIH-** Unsatisfactory/ Potential Injurious to Health; p-values < 0.01 means there is a significant relationship between the two tested independent variables.

### Genotypic and phenotypic characterization of *E. coli*, *Salmonella* spp. and *Listeria* spp. isolates

*E. coli* isolates were recovered from 91 out of the 98 samples. The remaining seven samples either did not contain *E. coli* or it was not possible to isolate *E. coli*, due to high levels of contamination with other microorganisms.

Antimicrobial resistant (AMR) *E. coli* was detected in 23/98 (23.5%) of the cheese samples, of which two (2/23; 8.7%) were multidrug resistant (MDR) (Table 3).

**Table 3:** Antimicrobial Resistance (AMR) of *E. coli* strains isolated from cheese samples

Cheese brand	Antimicrobial Resistance of *E. coli* isolate	Cheese brand	Antimicrobial Resistance of *E. coli* isolates
1E	AMP, AMC	5B	AMP, TET
2B	AMP, AMC, TET		AMC
	TET	5C	TET
	AMP, SMX	6A	TET
2C	AMP, AMC	6B	AMP, AMC
2D	*AMP, TET, CHL, SMX		TET
2E	AMP		AMC
2F	TET, TMP	7A	AMP, TET
2H	TET	8A	TET
3A	AMC	8B	TET
4C	TET, SMX		*AMP, TET, SMX
4E	AMP, AMC		

**AMP-** Ampicillin; **AMC-** Amoxicillin-Clavulanic Acid**; CHL-** Chloramphenicol; **TET-** Tetracycline; **TMP-** Trimethoprim; **SMX-** Sulfamethoxazole; * Multidrug resistant strain.

None of the 91 *E. coli* isolates presented any of the surveyed intestinal pathotype-specific virulence genes (*eae*, *aggR*, *aaiC*, *aatA*, *elt*, *esth*, *estp*, *ipaH*, *stx1* and *stx2*). WGS of the MDR (two) and hemolytic (one, that was antimicrobial susceptible) isolates, confirmed that they are all ExPEC.

The ExPEC hemolytic isolate belongs to ST14755 and to serotype O175:H16 (totally susceptible), while the two MDR ExPEC isolates belong to ST362; O4:H6 (resistant to ampicillin, tetracycline and sulfamethoxazole) and to ST155; OND:H11 (resistant to ampicillin, tetracycline, chloramphenicol and sulfamethoxazole).

*Salmonella* spp. strain, isolated from one of the cheeses, belongs to serotype Duisburg, and ST4046. Although containing one gene and one point mutation associated with antimicrobial resistance (aac(6')-Iaa; parC:p.T57S), this isolate was phenotypically susceptible to all the tested antimicrobials.

The four *Listeria monocytogenes* isolates belong to the following STs: ST1 (isolated from one of the 6B samples), ST5 (isolated from two samples of brand 1B) and ST7 (isolated from one of the 9A samples). The *Listeria innocua* isolates belong to ST492 (two of the 2G and one of the 2F samples) and ST603 (isolated from one of the 1C samples).

### Core-genome clustering analysis of *Listeria* spp. isolates

**Fig. 2:**
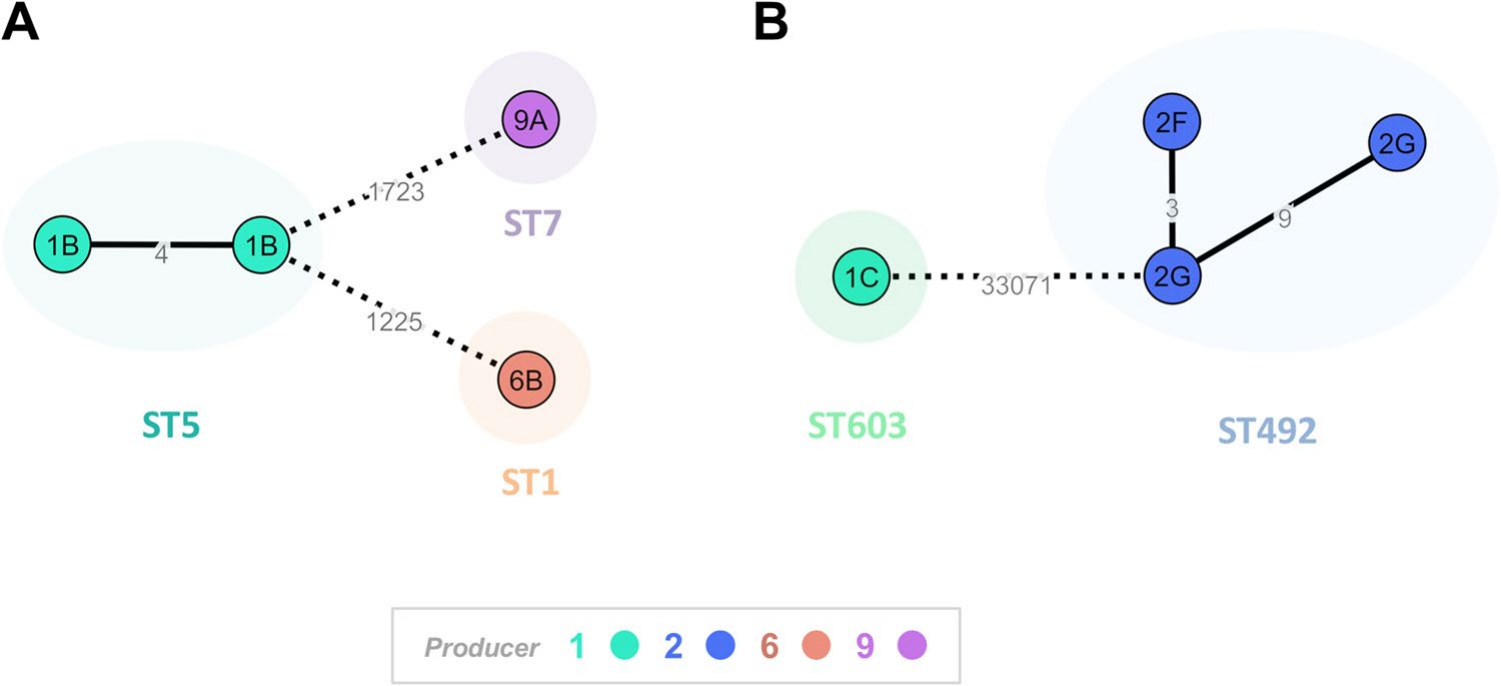
Core-genome clustering analysis of *Listeria* spp. isolates. (**A**) For *L. monocytogenes* (four isolates), the Minimum Spanning Tree (MST) was constructed based on the cgMLST 1748-loci Pasteur schema [38]. Each circle (node) contains the brand code and represents a unique allelic profile, with numbers on the connecting lines representing allelic distances (AD) between nodes. Straight and dotted lines reflect nodes linked with ADs below and above a threshold of seven ADs, which can provide a proxy to the identification of genetic clusters with potential epidemiological concordance [42]. (**B**) For *L. innocua* (four isolates), the MST was constructed based on a core-genome SNP-based alignment (comprising 95% of the *L. innocua* genome size) involving a total 33,081 variant sites. Each circle (node) contains the brand code designation and represents a unique SNP profile, with numbers on the connecting lines representing SNP distances between nodes. Straight and dotted lines reflect nodes linked with a SNP distance below and above a threshold of 15 SNPs. For both panels, data visualization was adapted from GrapeTree dashboard [41], with the node colors reflecting the producer and the surrounding shadows indicating the traditional seven-loci MLST classification

Despite the low number of isolates (four *per* species), core-genome clustering analysis of *L. monocytogenes* and *L. innocua* isolates was performed in order to assess the genetic relatedness among them and its potential correlation with cheese producer/brands (Fig. 2).

*L. monocytogenes* cgMLST revealed one cluster of closely related isolates (allelic distance = 4) comprising two ST5 isolates from brand 1B (producer 1). Similarly, *L. innocua* core-genome SNP-based alignment showed one cluster of ST492 isolates interconnected by ≤ 9 SNPs (enrolling 3 isolates, 2 from brand 2G and one from brand 2F), all also linked to the same producer (producer 2) (Fig. 2).

## Discussion

Cheeses may have physicochemical characteristics and shelf life favorable to the growth or survival of pathogenic bacteria [44, 45]. For instance, a pooled prevalence of *Listeria monocytogenes* (3.6%- sheep; 12.8%- goat), *Salmonella* spp. (5.9%- goat), *Staphylococcus aureus* (16.0%- goat) and STEC (2.8%- sheep; 4.3%- goat) in sheep and goat milk cheeses has been reported in a recent meta-analysis approach [46], which clearly demonstrates that these microorganisms are frequently detected in this type of foodstuffs. In particular, the production of cheeses free from *L. monocytogenes* may be a challenge for producers, since this pathogen is ubiquitous in nature, can grow at refrigeration temperatures, and forms biofilms, being difficult to eradicate from food contact surfaces in the production environment. In fact, many outbreaks associated with the consumption of cheeses have been caused by *L. monocytogenes* [47–51].

In this study, we have detected *L. monocytogenes* in four samples (4/98 = 4.1%) (3 with concentrations > 10^2^ cfu/g), and further found the presence of the indicator microorganism *L. innocua* in another four (4/98 = 4.1%). These results emphasize the need for monitoring good hygiene and manufacturing practices, as well as raw milk microbiological quality, in the context of Regulation (EC) N°852/2004 on the hygiene of foodstuffs [52], in the sector of Portuguese artisanal cheese industry.

The three identified *Listeria monocytogenes* sequence types (ST1, ST5, ST7) were already isolated, by others, in foodstuffs and associated to human disease [38, 53, 54]. All the *Listeria monocytogenes* detected STs were also already associated to Dairy Processing Facilities [55–57], with ST5 being described as a particularly persistent ST that might harbor specific efflux pump systems and heavy metal resistance genes that may possibly provide a higher tolerance to disinfectants [55].

The cgMLST analysis of *L. monocytogenes* isolates and SNP-analysis of *L. innocua* isolates suggest that, in this study, cheese contamination is potentially related with non-compliance with Good Hygiene Practices at producer level, since the two observed clusters (one for *L. monocytogenes* and one for *L. innocua*) are producer specific.

*S. aureus* is a common cause of bovine mastitis and is frequently detected in raw milk [58, 59]. In fact, in this study, we have found a statistically significant association between the microbiological safety classification of the samples regarding CPS enumeration and the use of cow’s milk in cheese production (Fisher-Freeman-Halton = 10.624, p = 0.004) (Table 2). Furthermore, enterotoxin-producing *S. aureus* exhibits a high osmotolerance, growing or surviving in cheeses with a_w_ levels as low as 0.86 (equivalent to about 20% NaCl), provided that all other conditions are optimal [60]. Thus, this bacterium may be a safety problem in raw milk cheeses, being already described as implicated in several outbreaks linked with milk and milk products [61]. In fact, in the present study, the detection of coagulase-positive Staphylococci > 10^4^ cfu/g in 6.1% (6/98) of the samples highlights for the importance of implementation of more efficient hygiene control measures during the process of cheese production and adequate selection of the raw materials. High counts of Staphylococci are also the main nonconformities found in Brazilian artisanal cheeses [62].

*Salmonella* spp. and pathogenic *E. coli* (in particular STEC) are also pathogens/hazards of concern when talking about cheese, being already detected in several outbreaks linked to the consumption of this category of foodstuffs [63–69].

In this research, *Salmonella* spp. was detected in one sample (1/98 = 1.0%). Serotype Duisburg, identified in the *Salmonella* spp. isolate, was recently associated to a foodborne outbreak that occurred in the United States of America in 2021, involving the consumption of cashew brie, a vegan alternative to Brie cheese [70].

Although no STEC isolates were found in the analyzed cheeses, we have detected the presence of Extraintestinal pathogenic *E. coli* (ExPEC) in three samples (3/98 = 3.1%), in accordance with our previous study performed on Alentejo raw milk cheeses [71]. ExPEC is recognized as the most common gram-negative pathogen in humans, causes most urinary tract infections (UTIs) in young healthy women, is the leading cause of bacteremia in adults, and is the second most common cause of neonatal meningitis [72]. This pathotype was also already detected in raw milk cheeses from various Latin America countries [73–75], and may potentially be transmitted to humans via food [76]. We have further found the presence of non-pathogenic *E. coli* > 10^4^ cfu/g in 16.3% of the samples (16/98), highlighting for potential non-conformities during processing along cheese production chain. ExPEC isolates were characterized as belonging to STs 14755, 155, and 362. *E. coli* ST155 and ST362 are highly widespread in nature, and have been isolated from wildlife, livestock, water, foodstuffs and humans, among other sources (Enterobase, https://enterobase.warwick.ac.uk/species/index/ecoli). *E. coli* ST155 has been described as a potential foodborne pathogen [77, 78]. *E. coli* ST 362 is known as a biofilm-producer [79] and was the dominant ST found in the calf ESBL-producing *E. coli* isolates from a dairy farm in Germany [80]. The ST14755, a novel sequence-type, was detected in a susceptible ExPEC hemolytic isolate, and belongs to serotype O175:H16.

Drug resistant microorganisms have become a threat to public health, with acute and chronic infections increasingly failing to respond to antimicrobial drugs. It is estimated that 700,000 people die because of resistant infections every year, and that by the year of 2050, 10 million deaths/ year can occur. This will result in a cumulative global economic loss of USD 100 trillion, due to the antimicrobial resistance effect [81]. It is well known that the unrestrained use of antibiotics to control disease in farm animals and in humans increases the number and frequency of antibiotic resistant bacterial isolates [82, 83]. In fact, in this study, 23.5% of the tested cheese samples presented *E. coli* isolates resistant to antimicrobials, with two of these strains being MDR. Antimicrobial resistance gene content of unprocessed animal products may potentially play a role in the acquisition of antimicrobial resistance of human pathogens [84].

The link of the above-mentioned pathogens with foodborne outbreaks related to cheese consumption, as well as the association between enhanced virulence and bacteria stress responses to cheese manufacturing, cheese matrix itself and storage conditions [85], highlights for the importance of the development of new instruments to control the presence of these bacteria during the process and, consequently, in the ready-to-eat end-product. Microbiological safety assessment of raw milk cheeses placed on the market is one of these instruments and may be essential to identify critical points along cheese production and to implement timely corrective measures, that may allow artisan cheesemakers to continue to produce their appreciated artisanal cheeses but with improved safety standards.

The results of this study clearly demonstrate that raw milk cheeses from Beira Baixa region, Portugal, may contain pathogenic and/or indicator microorganisms above the stipulated guideline limits, being some resistant to antimicrobials. It is essential not only to monitor the hygiene at the primary production (farm level), but also to implement corrective measures during cheese processing, to ensure control at the established critical points. In this study, we have found a statistical significant association between the microbiological safety classification of the samples and the use of cow’s milk in cheese production (Fisher-Freeman-Halton = 11.785, p = 0.001) (Table 2). This association also occurs when considering the microbiological safety classification of the samples regarding CPS and *E. coli* enumeration (Table 2), the two parameters that mostly contributed for the U/PIH classification of the samples. This result may point out for a potential safety critical point in Beira Baixa cheese production related with the hygiene at the cow’s milk production farms, and is a good example of how the microbiological safety assessment of the final product may be important to identify potential critical points along production.

The implementation and maintenance of training for the application of good manufacturing practices, and the constant monitoring of the quality of the production process in the Portuguese artisanal cheese industry, is of critical importance. To highlight also the importance of controlling the quality of the milk and to test environmental surfaces like food contact surfaces (e. g. cheese-making equipment, utensils, shelves for maturation/ripening) or non-food contact surfaces for preventing cross contamination with microbiological hazard thus improving the quality and safety of the final product.

### Supplementary Information

Below is the link to the electronic supplementary material.

Supplementary file1 (XLSX 27 kb)

## Data Availability

All supporting data and protocols have been provided within the article or through supplementary data files.
